# 515. Evolution of Treatment Patterns for Patients Hospitalized with COVID-19 in the United States

**DOI:** 10.1093/ofid/ofab466.714

**Published:** 2021-12-04

**Authors:** Kelly Zalocusky, Shemra Rizzo, Devika Chawla, Yifeng Chia, Tripthi Kamath, Larry Tsai

**Affiliations:** Genentech, Inc., South San Francisco, California

## Abstract

**Background:**

COVID-19 remains a threat to public health, with over 30 million cases in the US alone. As understanding of optimal patient care has improved, treatment guidelines have continued to evolve. This study characterized real-world trends in treatment for US patients hospitalized with COVID-19, stratified by whether patients required invasive ventilation.

**Methods:**

US patients diagnosed and hospitalized with COVID-19 between March 23 and December 31, 2020, in the Optum de-identified COVID-19 electronic health record (EHR) data set were identified. Both drug and procedure codes were used to ascertain medications, and both procedure and diagnostic codes were used to detect invasive ventilation during hospitalization. Medication trends were estimated by computing proportions of hospitalized patients receiving each drug weekly during the study period.

**Results:**

In this cohort of 71,366 hospitalized patients, the largest observed change in care was related to chloroquine/hydroxychloroquine (HCQ) (Figure). HCQ usage peaked at 87% of patients receiving invasive ventilation (54% without ventilation) in the first week of this study (March 23-29), but declined to < 5% of patients, regardless of ventilation status, by the end of May. In contrast, dexamethasone usage was 10% at baseline in patients receiving ventilation (1% without ventilation) and increased to a steady state of >85% of patients receiving ventilation ( >50% without ventilation) by the end of June. Similarly, remdesivir usage increased sharply from a baseline of 2% of patients and continued to rise to a peak of 79% of patients receiving invasive ventilation (44% without ventilation) in November before declining.

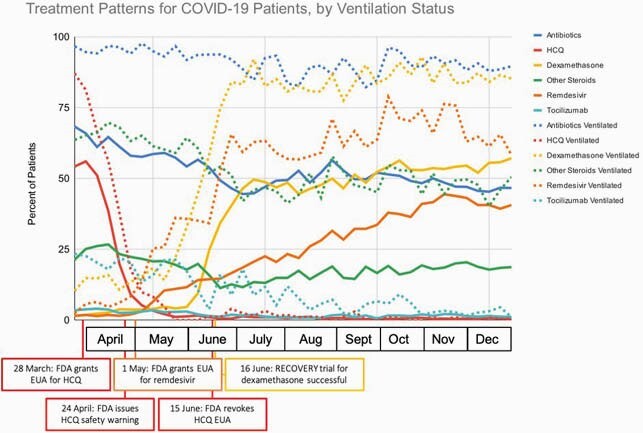

**Conclusion:**

Meaningful shifts in treatments for US patients hospitalized with COVID-19 were observed from March through December 2020. A dramatic decline was observed for HCQ use, likely owing to safety concerns, while usage of dexamethasone and remdesivir increased as evidence of their efficacy mounted. Across medications, usage was substantially more prevalent among patients requiring invasive ventilation compared with patients with less severe cases.

**Disclosures:**

**Kelly Zalocusky, PhD**, **F. Hoffmann-La Roche Ltd.** (Shareholder)**Genentech, Inc.** (Employee) **Shemra Rizzo, PhD**, **F. Hoffmann-La Roche Ltd.** (Shareholder)**Genentech, Inc.** (Employee) **Devika Chawla, PhD MSPH**, **F. Hoffmann-La Roche Ltd.** (Shareholder)**Genentech, Inc.** (Employee) **Yifeng Chia, PhD**, **F. Hoffmann-La Roche Ltd** (Shareholder)**Genentech, Inc.** (Employee) **Tripthi Kamath, PhD**, **F. Hoffmann-La Roche Ltd** (Shareholder)**Genentech, Inc.** (Employee) **Larry Tsai, MD**, **F. Hoffmann-La Roche Ltd** (Shareholder)**Genentech, Inc.** (Employee)

